# HDAC3-driven immunometabolic reprogramming in type 2 diabetes and its complications: from enzymatic deacetylation to non-enzymatic scaffolding

**DOI:** 10.3389/fimmu.2026.1890366

**Published:** 2026-09-23

**Authors:** Nianxin Kang, Qiqi Zhang, Xiangyuan Zhang, Wenhao Cheng, Zezheng Gao, Tingting Bao, Linhua Zhao

**Affiliations:** 1 Institute of Metabolic Diseases, Guang’anmen Hospital, China Academy of Chinese Medical Sciences, Beijing, China; 2 Graduate College, Beijing University of Chinese Medicine, Beijing, China; 3 Department of Pharmacy, China-Japan Friendship Hospital, Beijing, China; 4 Department of Traditional Chinese Medicine, The Affiliated Hospital of Qingdao University, Qingdao, China

**Keywords:** diabetic complications, epigenetic regulation, histone deacetylase 3, immunometabolism, type 2 diabetes

## Abstract

Type 2 diabetes mellitus (T2DM) is driven by the intricate interplay between metabolic dysregulation and chronic inflammation. However, the comprehensive epigenetic mechanisms linking immune dysfunction to metabolic abnormalities remain incompletely understood. Histone deacetylase 3 (HDAC3), which possesses both deacetylase catalytic activity and non-enzymatic scaffold functions, has emerged as a critical regulator of immunometabolic homeostasis. Nevertheless, its coordinated pathogenic roles in T2DM and its multi-organ complications have not been fully elucidated. This review comprehensively integrates the regulatory mechanisms of HDAC3 in β-cell dysfunction, insulin resistance, mitochondrial impairment, and chronic inflammation, highlighting its pivotal role in the initiation and progression of T2DM. We further systematically summarize the shared pathogenic mechanisms mediated by HDAC3 in diabetic cardiomyopathy, nephropathy, retinopathy, encephalopathy, and impaired wound healing. In addition, we focus on precision therapeutic strategies targeting HDAC3, with particular emphasis on recent advances and translational challenges involving microRNAs (miR) and naturally derived HDAC3 inhibitors. Collectively, this review identifies HDAC3 as a central epigenetic hub underlying T2DM and its complications, providing novel mechanistic insights and a theoretical foundation for the development of precision-targeted therapies for diabetes and its associated complications.

## 1 Introduction

Diabetes mellitus is a heterogeneous syndrome characterized by hyperglycemia and can be classified into type 1 diabetes mellitus (T1DM), type 2 diabetes mellitus (T2DM), gestational diabetes mellitus, and specific types of diabetes. T2DM is a non-autoimmune metabolic disorder characterized by systemic insulin resistance (IR), chronic low-grade inflammation, and progressive deterioration of pancreatic β-cell function (1). In 2021, approximately 537 million people worldwide were living with diabetes, and more than 6.7 million deaths among individuals aged 20–79 years were attributed to diabetes-related diseases (2). T2DM accounts for approximately 96% of all diabetes cases and represents one of the most prevalent noncommunicable chronic diseases threatening global public health. Persistent hyperglycemia can induce target organ damage and lead to multiple complications (3). Accumulating evidence suggests that persistent immunometabolic dysregulation, characterized by reciprocal interactions between chronic inflammation and metabolic remodeling, represents a common pathogenic mechanism underlying T2DM and its multiple complications (4, 5). Given that current glucose-lowering therapies are often insufficient to halt the progression of diabetic complications, elucidating the molecular mechanisms governing immunometabolic dysregulation and identifying upstream regulatory targets have become major priorities for developing more effective therapeutic strategies.

During the development and progression of diabetes, the genetic architecture itself is not directly altered but is dynamically regulated through epigenetic mechanisms. In this process, genes and histones undergo chemical modifications that selectively influence protein expression, thereby contributing to the onset and progression of diabetes and its complications. Emerging evidence further suggests that chronic inflammatory signaling reshapes cellular metabolic programs through epigenetic remodeling, thereby contributing to persistent immunometabolic dysfunction in T2DM (6). Acetylation and deacetylation are considered critical regulatory events. Increasing evidence indicates that epigenetic regulators, particularly histone deacetylases (HDACs), play central roles in controlling gene expression programs associated with inflammation, metabolism, and tissue homeostasis (7). Among class I HDAC family members, HDAC3 possesses several unique biological features, including dynamic nucleocytoplasmic shuttling, dependence on the NCoR/SMRT co-repressor complex for full catalytic activity, and the ability to exert both enzymatic deacetylase-dependent and non-enzymatic scaffolding functions. These properties enable HDAC3 to coordinate transcriptional regulation, metabolic adaptation, and inflammatory signaling in a context-dependent manner (8). Clinical studies have demonstrated that HDAC3 activity and HDAC3 mRNA expression in peripheral blood mononuclear cells from patients with T2DM are positively correlated with pro-inflammatory responses, poor glycemic control, and insulin resistance (9). In addition, HDAC3 is widely expressed in metabolic organs, including the liver, adipose tissue, skeletal muscle, pancreas, and intestine, as well as in immune cells such as macrophages, thereby positioning HDAC3 as a critical nexus linking immune signaling and metabolic regulation (10).

Aberrant activation of HDAC3 drives metabolic reprogramming, impairs insulin signaling, amplifies inflammatory responses, and contributes to organ-specific dysfunction. Collectively, these processes promote the onset and progression of T2DM and its major complications, including diabetic cardiomyopathy, nephropathy, retinopathy, and neuropathy. Nevertheless, several critical questions remain unresolved, particularly regarding the context-dependent effects of HDAC3 within distinct transcriptional and metabolic networks and the relative contributions of its enzymatic versus scaffold functions to disease progression.

Although previous reviews have summarized the roles of HDAC3 in oxidative stress, chronic organ injury, and inflammatory diseases (11), or broadly discussed the involvement of HDAC family members in diabetes (12), a comprehensive synthesis of how the dual functional modes of HDAC3 contribute to the pathogenesis of T2DM and its complications remains lacking. Compared with previous reviews, the present review provides three major advances. First, we establish HDAC3-mediated immunometabolic reprogramming as a unified conceptual framework to interpret the initiation and progression of T2DM and its complications across multiple metabolic organs. Second, we systematically distinguish the enzymatic deacetylase-dependent and non-enzymatic scaffolding functions of HDAC3, highlighting their distinct molecular mechanisms, biological consequences, and pathological significance in T2DM and its complications. Third, we comprehensively integrate and comparatively evaluate current HDAC3-targeted therapeutic strategies, including selective small-molecule inhibitors, microRNA-based interventions, and naturally derived compounds, with emphasis on their mechanisms of action, translational potential, and current limitations. Collectively, the overall conceptual framework summarizing the roles of HDAC3 in T2DM and its complications, together with current therapeutic strategies, is illustrated in **Figure 1**. By integrating molecular mechanisms with therapeutic perspectives, this review aims to provide a conceptual roadmap for future mechanistic investigations and the development of HDAC3-targeted interventions for T2DM and its complications.

**Figure 1:**
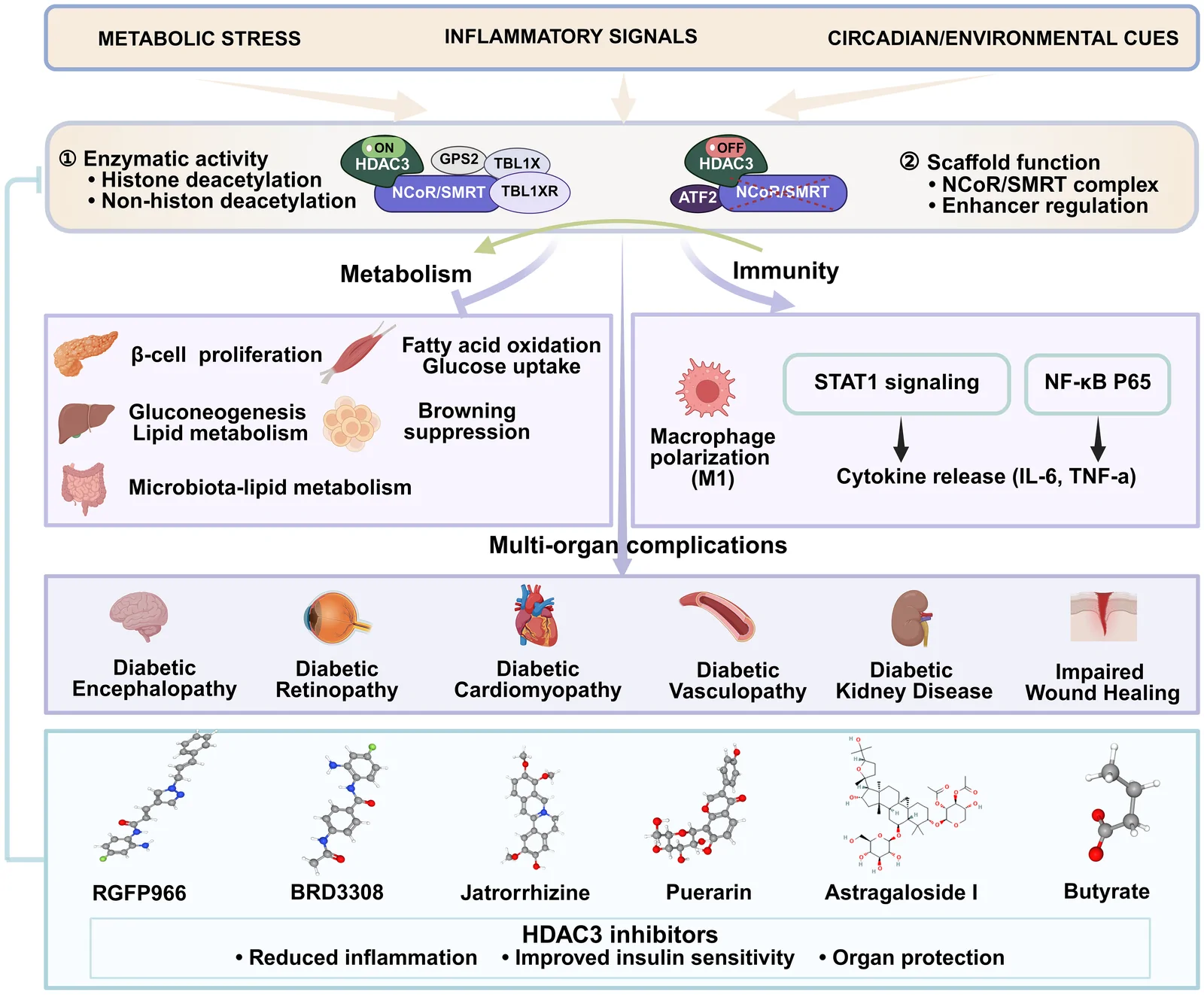
Graphical abstract illustrating the multifaceted roles of HDAC3 in the pathogenesis of T2DM and its complications. HDAC3 possesses dual functional modes, including enzymatic deacetylase-dependent activity and non-enzymatic scaffolding functions, which collectively regulate transcriptional programs, metabolic adaptation, inflammatory signaling, oxidative stress, and cellular homeostasis. Aberrant HDAC3 activation contributes to insulin resistance and promotes the development of diabetic complications, through coordinated immunometabolic dysregulation. Current HDAC3-targeted therapeutic strategies, including selective inhibitors, microRNA-based interventions, and naturally derived compounds, may alleviate disease progression by modulating HDAC3-associated signaling pathways. This graphical abstract summarizes the integrated conceptual framework discussed throughout this review.

## 2 HDAC family: classification and functional landscape

In eukaryotes, DNA is wrapped around a histone octamer, which consists of two H2A-H2B dimers and one (H3-H4)_2_ tetramer, forming nucleosomes as the basic chromatin unit (13). Epigenetic modifications serve as molecular marks that distinguish distinct chromatin states. Dysregulated histone acetylation can disrupt gene expression programs and contribute to disease onset and progression (14). Histone acetylation homeostasis is tightly controlled by the opposing activities of histone acetyltransferases (HATs) and HDACs (15). HATs catalyze the acetylation of lysine residues on histone tails or non-histone substrates. By neutralizing the positive charge of lysine residues on histone tails, HAT-mediated acetylation relaxes nucleosomal structure, increases chromatin accessibility, and facilitates the recruitment of RNA polymerase II, thereby promoting transcriptional activation (16). Conversely, HDACs remove acetyl groups, restoring the positive charge on lysine side chains, tightening chromatin structure, and restricting RNA polymerase access, ultimately suppressing transcription (**Figure 2**).

**Figure 2:**
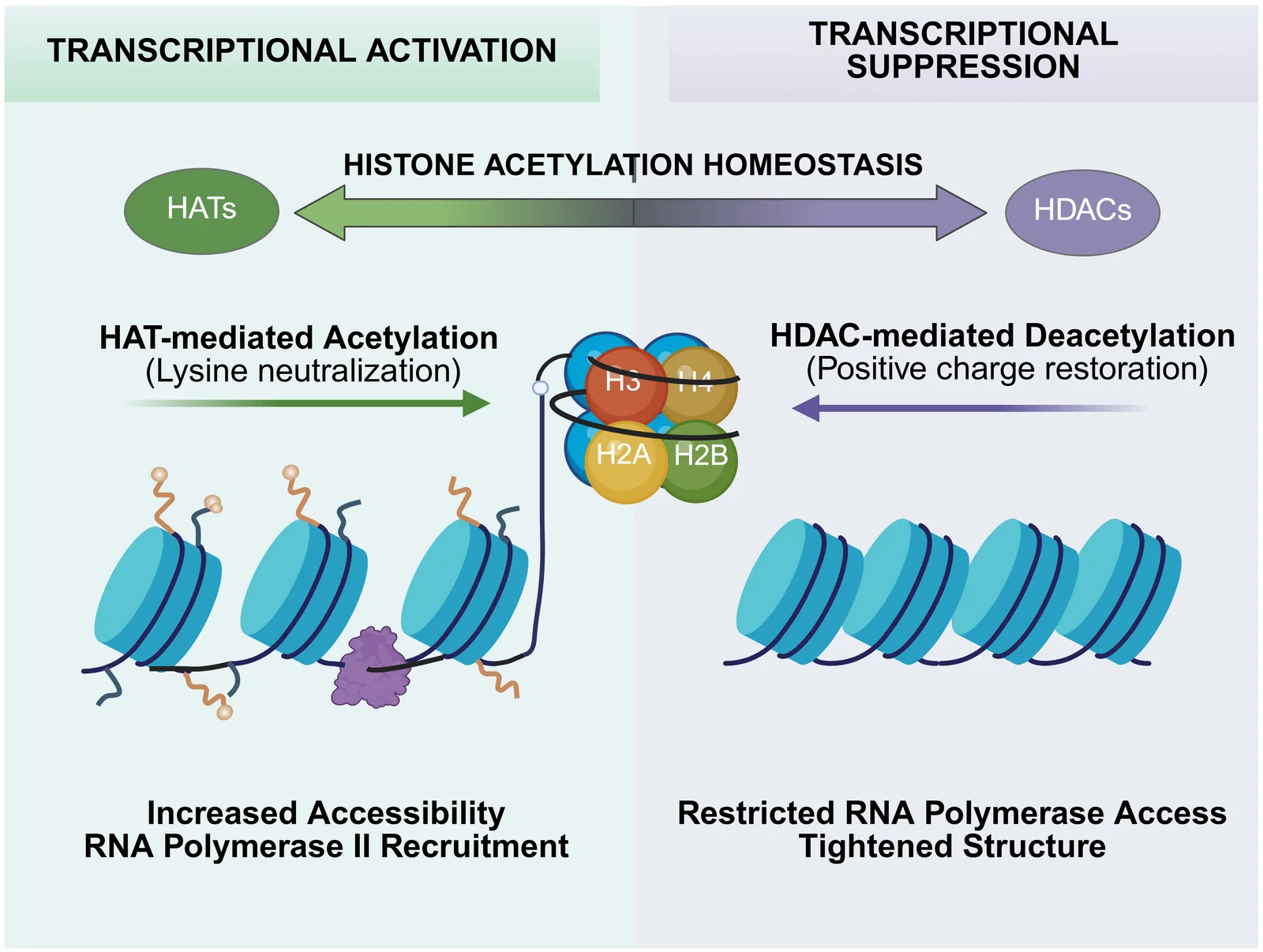
Schematic representation of histone acetylation homeostasis in chromatin regulation. The left part shows the transcriptional activation process mediated by histone acetyltransferases (HATs). HATs catalyze the acetylation of lysine residues of core histones H2A, H2B, H3, and H4. Acetylation neutralizes the positive charge of histone tails, weakening the electrostatic attraction between histones and DNA, unfolding nucleosomes into open chromatin and facilitating the recruitment of RNA polymerase II to activate gene transcription. The right part displays transcriptional repression mediated by histone deacetylases (HDACs). HDACs eliminate acetyl groups from histone lysine sites, restoring the positive charge of histone tails, tightening the binding between histones and DNA to form condensed chromatin, and blocking the access of RNA polymerase II to inhibit gene expression. The antagonistic catalytic activities of HATs and HDACs jointly maintain the dynamic homeostasis of histone acetylation, which serves as a core epigenetic switch governing global transcriptional programs.

18 mammalian HDACs have been identified to date and are grouped into four classes based on sequence homology and cofactor dependency (**Figure 3A**). Class I HDACs include HDAC1, HDAC2, HDAC3, and HDAC8; class II HDACs are subdivided into class IIa (HDAC4, 5, 7, 9) and class IIb (HDAC6, 10); class IV comprises only HDAC11; and class III HDACs refer to the sirtuin family (SIRT1-SIRT7) (17). These HDACs exhibit distinct subcellular localizations and diverse biological functions (**Figure 3B**) (18). HDAC1, HDAC2, and HDAC8 are predominantly nuclear. HDAC3, HDAC4, HDAC5, HDAC6, HDAC7, HDAC9, HDAC10, and HDAC11 localize to both the nucleus and the cytoplasm, although HDAC3 and HDAC11 are mainly nuclear, whereas HDAC6 and HDAC10 are largely cytoplasmic. HDAC4/5/7/9 share similar nucleocytoplasmic shuttling mechanisms: upon phosphorylation, they translocate to the cytoplasm and interact with chromosomal region maintenance 1 (CRM1) (19). Catalytically, class I, II, and IV HDACs function as zinc-dependent enzymes (20), whereas class III HDACs require nicotinamide adenine dinucleotide (NAD^+^) as a cofactor (21).

**Figure 3:**
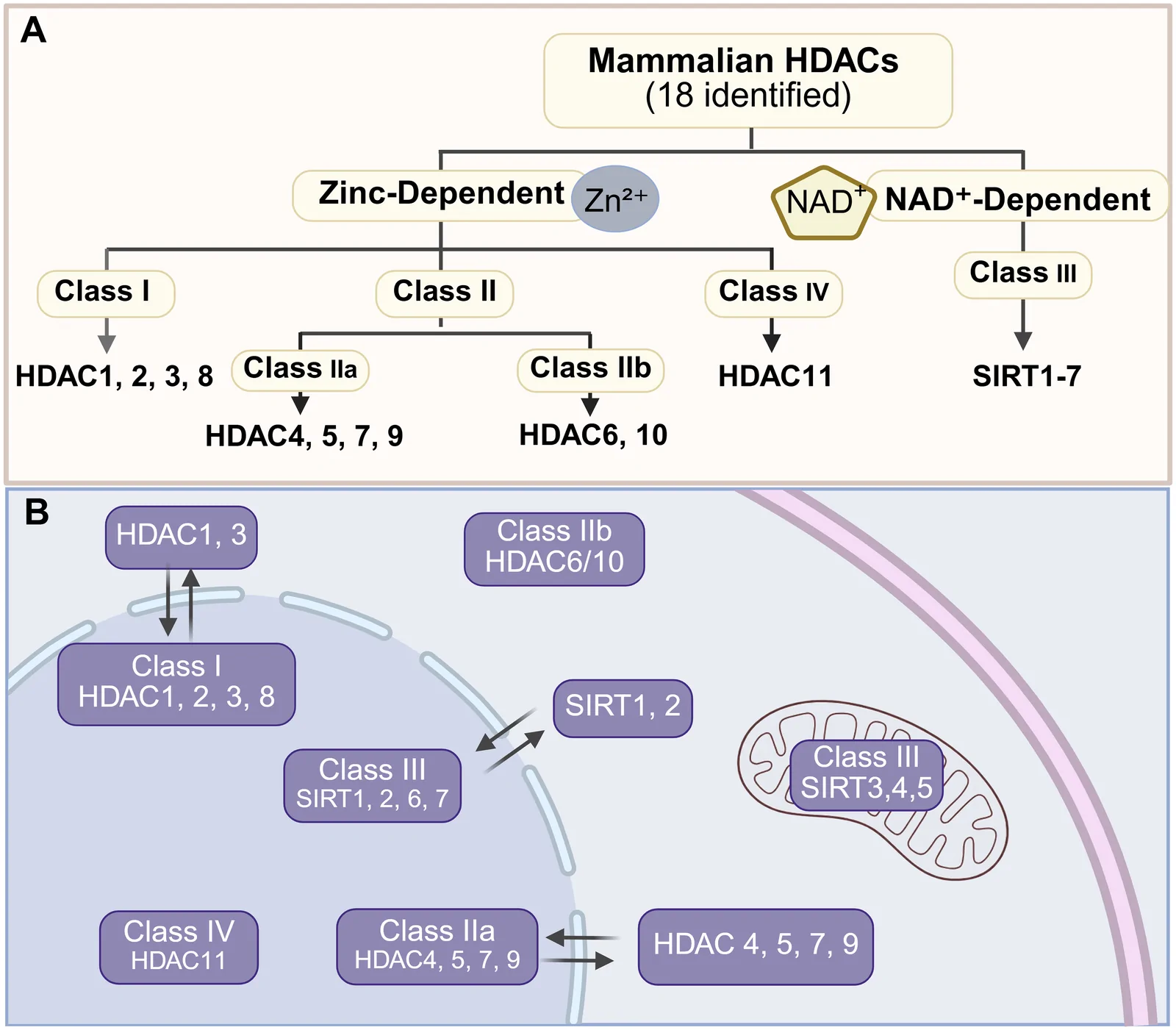
Classification and subcellular localization of mammalian HDACs. **(A)** shows the taxonomic grouping of the 18 identified mammalian HDAC isoforms divided by catalytic cofactor requirements and sequence homology. All HDACs are first split into zinc-dependent and NAD^+^-dependent branches. Zinc-dependent HDACs contain Class I (HDAC1, 2, 3, 8), Class II (subdivided into Class IIa: HDAC4, 5, 7, 9 and Class IIb: HDAC6, 10), and the single-member Class IV (HDAC11). The NAD^+^-dependent branch corresponds to Class III sirtuins (SIRT1-7). **(B)** illustrates the characteristic subcellular distribution patterns of each HDAC class within mammalian cells. Class I HDAC1, 2, 3, 8 mainly reside in the nucleus, while HDAC1 and HDAC3 dynamically shuttle between the nucleus and cytoplasm. Class IIa HDAC4, 5, 7, 9 and Class IIb HDAC6, 10 undergo bidirectional nucleocytoplasmic transport. Class IV HDAC11 is primarily localized to the nucleus. Class III sirtuins display widespread compartmentalization: SIRT1/2/6/7 are detected in the nucleus, SIRT1/2 can translocate to the cytoplasm, and SIRT3/4/5 exclusively localize to mitochondria.

Aside from HDAC8, which operates independently, class I HDACs require assembly into multiprotein complexes as a fundamental prerequisite for exerting their enzymatic functions. HDAC3 forms stable complexes with nuclear receptor (NR) corepressors, including nuclear receptor corepressor 1 (NCoR1) and silencing mediator of retinoid and thyroid hormone receptors (SMRT, also known as NCoR2) (22). Although *in vitro* studies have shown that other class I and class II HDACs can associate with the NCoR/SMRT complexes (23, 24), HDAC3 is the only HDAC known to bind NCoR/SMRT endogenously *in vivo*. Importantly, HDAC3 requires direct physical interaction with the deacetylase-activation domains (DADs) of NCoR and SMRT to achieve full catalytic activity-a structural requirement that underlies its unique role in chromatin remodeling and transcriptional regulation (25–27) (**Figure 4**). Notably, global deletion of HDAC3 results in early embryonic lethality (28), indicating that HDAC3 exerts essential functions beyond its catalytic activity, including non-enzymatic scaffold roles.

**Figure 4:**
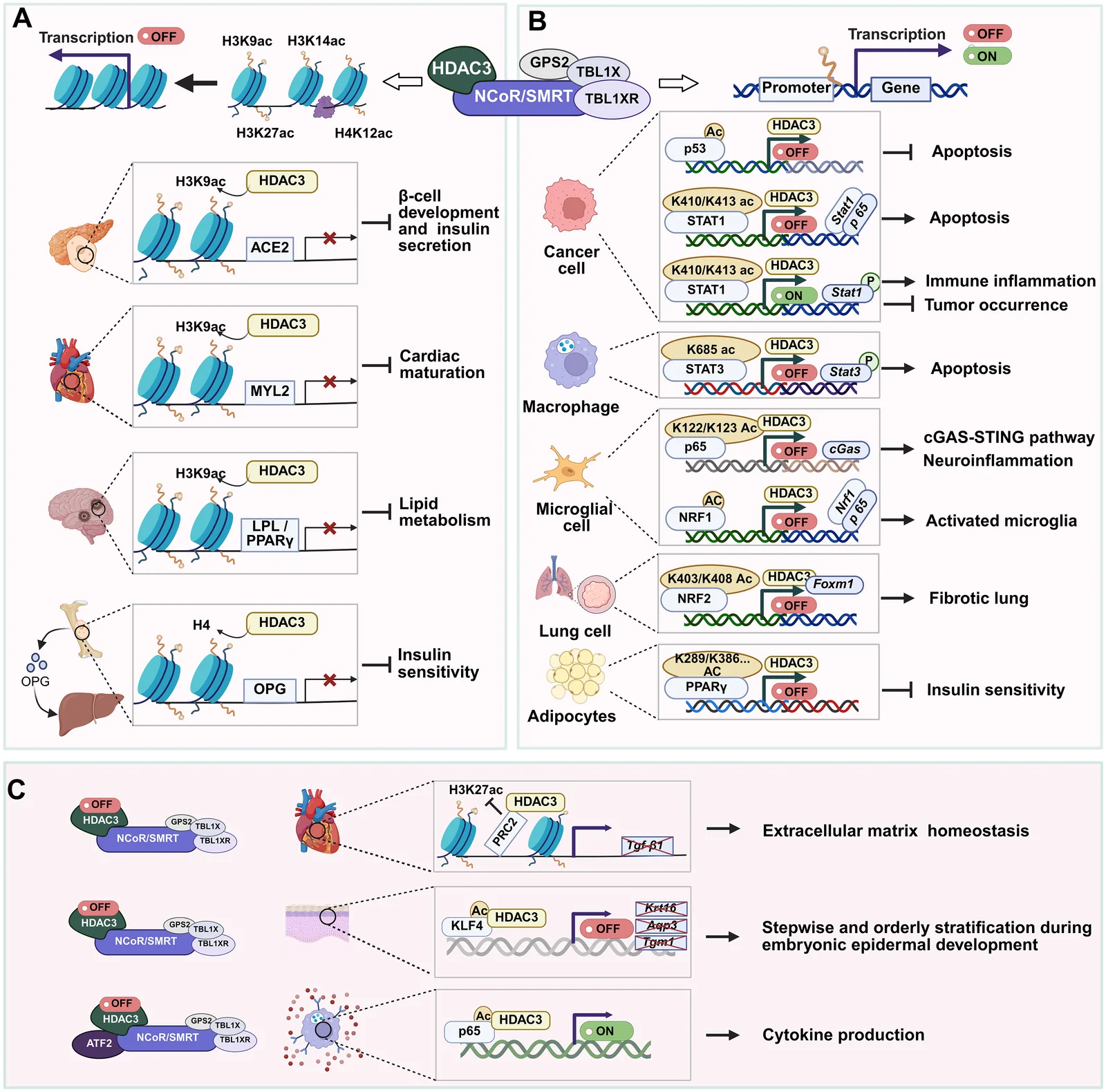
Enzymatic and non-enzymatic functions of HDAC3 in transcriptional regulation. **(A)** shows HDAC3’s histone deacetylase enzymatic function. In combination with the NCoR/SMRT corepressor complex, HDAC3 removes acetyl modifications from core histones, condenses chromatin and inhibits gene transcription, thereby negatively regulating pancreatic β-cell function, cardiac maturation, lipid metabolism and insulin sensitivity across multiple metabolic organs. **(B)** depicts HDAC3-mediated deacetylation of non-histone transcription factors in various somatic and immune cells. By erasing acetyl groups on key transcription factors, HDAC3 modulates cell apoptosis, inflammatory response, tumor progression, pulmonary fibrosis and systemic metabolic balance. **(C)** introduces the catalysis-independent scaffolding function of HDAC3. As a component of corepressor complexes, HDAC3 regulates target gene expression without deacetylase activity, participating in cardiac extracellular matrix homeostasis, embryonic epidermal development and inflammatory cytokine secretion.

## 3 HDAC3 functions: enzymatic and non-enzymatic functional modes

### 3.1 Dual functional modes of HDAC3 in transcriptional regulation

HDAC3 participates in transcriptional regulation through two distinct functional modes. In its enzymatic mode, it exerts deacetylase activity (DA) in an NCoR/SMRT-dependent manner, promoting chromatin compaction and transcriptional repression. In its non-enzymatic mode, HDAC3 acts as a scaffold within transcriptional complexes, stabilizing the assembly of transcription factors and coregulators independently of its catalytic activity. This dual functional modality underlies the broad involvement of HDAC3 in immunometabolic regulation. These regulatory effects collectively position HDAC3 as a key mediator linking immune signaling to metabolic transcriptional programs (**Figure 4**).

NRs, as signal-dependent transcription factors, integrate developmental, hormonal, environmental, and nutrient cues and relay them to the genome, thereby functioning as transcriptional switches (29, 30). Ligand binding induces conformational rearrangements in nuclear receptors, dictating the selective recruitment of coactivators or corepressors (31, 32). Beyond nuclear receptors, other transcription factors also sense and integrate hormonal, metabolic, and environmental signals to recruit diverse cofactors to specific genomic loci. HDAC3-containing NR corepressor complexes, including NCoR and SMRT, bind to unliganded NRs to mediate transcriptional repression and, in certain contexts, can also indirectly contribute to transcriptional activation. These complexes establish HDAC3 as a central epigenetic regulator.

### 3.2 HDAC3 enzymatic activity in chromatin regulation: histone deacetylation

The canonical function of HDAC3 is to remove acetyl groups from histone tails, thereby promoting chromatin compaction and repressing transcription (33). HDAC3 primarily targets lysine residues on histones H3 and H4, including H3 lysine 9 (H3K9), H3K14, H3K27, H4K5, and H4 lysine 12 (H4K12) (34).

In pancreatic β-cells, HDAC3 upregulation leads to reduced H3K9ac, H3K14ac, and H3K27ac levels at the angiotensin-converting enzyme 2 (ACE2) promoter, impairing β-cell development and insulin secretion (35). During cardiac development, HDAC3 reduces H3K9ac at the Myosin Light Chain 2 (Myl2) promoter, resulting in defective cardiac maturation in offspring (36). In neurodegenerative disorders, the accumulation of amyloid-β (Aβ) enhances HDAC3 catalytic activity, leading to reduced H3K9ac at the promoters of lipoprotein lipase (LPL) and peroxisome proliferator-activated receptor gamma (PPARγ). This epigenetic repression disrupts lipid metabolism and contributes to the pathogenesis of Alzheimer’s disease (AD) (37).

Within the bone–liver metabolic axis, osteoprogenitor cells increase osteoprotegerin (OPG) secretion, which alleviates hepatic inflammation by suppressing receptor activator of nuclear factor κB ligand (RANKL) signaling and improves systemic insulin resistance. HDAC3 counteracts this protective mechanism by removing H4 acetylation at the OPG promoter, repressing OPG transcription, diminishing bone-derived anti-inflammatory signals, and ultimately leading to impaired insulin sensitivity (38) (**Figure 4A**).

### 3.3 HDAC3 enzymatic activity toward non-histone transcription factor*s*

Beyond histones, HDAC3 also deacetylates a wide range of transcription factors, thereby directly modulating their activity, stability, or DNA-binding capacity. Known substrates include p53, nuclear factor kappa B (NF-κB), nuclear factor erythroid 2-like 1/2 (Nrf1/2), signal transducer and activator of transcription proteins (STAT), and PPARγ.

The tumor suppressor p53 is a central mediator of cellular signaling and stress responses, orchestrating the transcription of genes involved in cell-cycle arrest, senescence, and apoptosis (39). It was the first non-histone protein shown to undergo acetylation (40). Acetylation of lysine residues located within its DNA-binding domain (DBD) and C-terminal domain (CTD) enhances DNA binding and promotes transcriptional activation of its target genes (41). HDAC3 suppresses p53 acetylation, and phosphorylation of HDAC3 by PINK1 further strengthens the HDAC3-p53 interaction, resulting in reduced p53 acetylation and diminished transcriptional activity (42, 43).

NF-κB is a key regulator of inflammatory responses, and its p65 subunit contains multiple reversible acetylation sites, including Lys122, Lys123 (44), Lys218, Lys221, and Lys310 (45). Acetylation at Lys122/123 promotes nuclear export and reduces DNA-binding affinity (46). HDAC3 deacetylates p65 at Lys122 in the cytoplasm and also interacts with nuclear p65 to enhance DNA binding, thereby activating the cGAS-STING-NF-κB pathway and amplifying inflammatory responses (47).

Nrf1 is a major transcription factor that maintains intracellular redox homeostasis and is essential for cellular resistance to endogenous and exogenous stressors (48). Chen and colleagues (49) reported that HDAC3 deacetylates Nrf1 at Lys105 and Lys139, thereby enhancing the activity of the p65 gene promoter and promoting the release of tumor necrosis factor alpha (TNF-α) and interleukin-1 beta (IL-1β) in microglia. Nrf2 functions as a redox-responsive master regulator of antioxidant gene expression and plays a central role in safeguarding cells against oxidative injury elicited by metabolic, inflammatory, or environmental stressors. Unlike the well-characterized regulation of Nrf1, HDAC3 has been reported to negatively regulate the antioxidant activity of Nrf2, as reflected by the inverse association between HDAC3 enzymatic activity or protein abundance and Nrf2-dependent antioxidant responses (50–52), although the precise deacetylation sites targeted by HDAC3 remain to be defined.

PPARγ plays a central role in regulating glucose and fatty-acid metabolism. The transcriptional activity of PPARγ is controlled by its ligands, which determine its interactions with coactivators and corepressors. In the absence of ligand stimulation, PPARγ associates with the HDAC3-containing corepressor complex formed by SMRT or NCoR, thereby suppressing its transcriptional activity and reducing insulin sensitivity, potentially through HDAC3-mediated targeting of four acetylated lysine residues in PPARγ (Lys289, Lys386, Lys462, and Lys466) (53).

STAT proteins constitute a family of latent cytoplasmic transcription factors that are activated through tyrosine phosphorylation mediated by members of the Janus kinase (JAK) family in response to a wide range of cytokines and growth factors (54). Acetylation of STAT1 attenuates its tyrosine phosphorylation and activation, whereas deacetylated STAT1 is less readily recognized by the phosphatase TCP45, thereby stabilizing its phosphorylated form and increasing p-STAT1 levels (55, 56). HDAC3 has been reported to mediate this effect by targeting the key lysine residues Lys410 and Lys413 (57). HDAC3 deacetylates STAT1, thereby promoting its phosphorylation and activating the AIM inflammasome (58). Previous studies have shown that acetylated STAT1 interacts with NF-κB p65, enhancing p65 DNA binding and nuclear localization and ultimately promoting apoptosis (59).

STAT3 is a well-known regulator of cell growth and proliferation, and its activity is modulated by post-translational modifications, primarily acetylation and phosphorylation (60). The major regulatory residues include phosphorylation at Tyr705 (p-Tyr705), phosphorylation at Ser727 (p-Ser727), and acetylation at Lys685 (ac-Lys685). Acetylation at Lys685 occurs upstream of Tyr705 phosphorylation and is required for subsequent STAT3 activation. In contrast to its effect on p-STAT1, HDAC3-mediated deacetylation of STAT3 at Lys685 suppresses the transcription of STAT3 target genes by reducing STAT3 phosphorylation and activation, thereby inhibiting downstream HIF-1α expression (61, 62).

Taken together, these studies suggest that although HDAC3 catalyzes the same deacetylation reaction on both histone and non-histone substrates, the biological outcomes are highly substrate-specific. This substrate-dependent variability likely arises from several factors, including the structural context of the acetylated lysine residues, the distinct functional domains in which these residues reside, differential interactions with cofactors or protein complexes, and the integration of each substrate into specific cellular signaling networks. For example, acetylation of STAT3 enhances its DNA-binding capacity, transcriptional activity, and nuclear localization, whereas HDAC3-mediated deacetylation suppresses the transcription of STAT3 target genes (63). By comparison, acetylation of STAT1 attenuates its signaling output (57, 64). HDAC3 also deacetylates Lys122 of the NF-κB p65 subunit in the cytoplasm to potentiate inflammatory responses, while deacetylation of p53 diminishes its pro-apoptotic activity. At the regulatory level, HDAC3-mediated deacetylation of histones primarily modulates chromatin accessibility, thereby exerting broad epigenomic effects. In contrast, deacetylation of non-histone transcription factors directly fine-tunes their phosphorylation, DNA-binding capacity, nuclear localization, and transcriptional activity in a substrate- and lysine-specific manner. Collectively, these layers of regulation cooperate to determine whether specific transcriptional programs are activated or repressed, highlighting the substrate-specific functional outcomes of HDAC3 activity (**Figure 4B**).

### 3.4 Non-enzymatic (scaffold) functions of HDAC3

Accumulating evidence indicates that HDAC3 possesses significant transcriptional regulatory capacity independent of its catalytic activity. For example, the Y298F mutation abolishes HDAC3 deacetylase activity, yet the mutant protein partially rescues hepatic steatosis and suppresses lipogenic gene expression. In mice carrying mutations in the DAD of either NCoR or SMRT, HDAC3 enzymatic activity is abolished while protein expression remains intact (65).

In lipopolysaccharide (LPS)-stimulated macrophages, HDAC3 regulates two distinct classes of genes. Deacetylase activity-dependent (DA-dependent) genes require its enzymatic function and are generally associated with anti-inflammatory effects. In contrast, deacetylase activity-independent (DA-independent) genes rely on the structural presence of HDAC3 to promote enhancer RNA (eRNA) production and increase H3K27 acetylation, thereby facilitating inflammatory gene expression. In Y298F mutant mice, HDAC3 fails to facilitate ATF3-dependent transcriptional repression of DA-dependent inflammatory genes, but retains its pro-inflammatory enhancer scaffold function on DA-independent genes (66). The findings underscore that the non-enzymatic functions of HDAC3 are essential for maintaining immune homeostasis.

Collectively, HDAC3 regulates chromatin accessibility and transcription factor activity through its enzymatic function, while its non-enzymatic scaffold role shapes transcriptional complexes and enhancer activity. This dual mode of regulation establishes HDAC3 as a central integrator linking epigenetic modifications, inflammatory signaling, and metabolic homeostasis. Importantly, recent studies have begun to reveal the physiological relevance of HDAC3’s non-enzymatic functions. In the heart, HDAC3 functions as a deacetylase-independent epigenetic silencer of transforming growth factor-β1 (TGF-β1) by recruiting enhancer of zeste homolog 2 (EZH2) as part of the NCoR complex. Loss of HDAC3 increases TGF-β bioavailability, promotes persistent mesenchymal activation, induces aberrant endothelial-to-mesenchymal transition, and disrupts extracellular matrix homeostasis, as observed in human semilunar valve pathologies (67). Similarly, in embryonic epidermis, the non-enzymatic function of HDAC3, stabilized by NCoR and SMRT, cooperates with KLF4 to repress the aberrant expression of differentiation-associated genes, ensuring proper epidermal stratification (28). These findings provide direct evidence that HDAC3’s non-enzymatic scaffold function can regulate developmental processes. Nonetheless, the precise physiological roles of the non-enzymatic functions of HDAC3 remain incompletely understood, and whether their dysregulation directly contributes to disease pathogenesis warrants further investigation (**Figure 4C**). Although these studies provide important mechanistic insights into the non-enzymatic scaffolding functions of HDAC3, the current evidence is derived predominantly from cellular and experimental models. Whether these mechanisms operate in human diabetic tissues remains largely unknown and warrants further investigation.

Taken together, the evidence discussed above highlights HDAC3 as a central immunometabolic regulator whose biological functions extend far beyond histone deacetylation. In addition to its enzymatic activity, HDAC3 exerts non-enzymatic scaffolding functions by interacting with transcription factors, co-regulatory complexes, and signaling molecules, thereby shaping gene expression programs in a context-dependent manner. These dual functional modes enable HDAC3 to couple nutrient sensing, metabolic adaptation, and inflammatory responses across multiple tissues. As these regulatory mechanisms become dysregulated, HDAC3 progressively reprograms tissue-specific transcriptional networks that govern metabolic homeostasis. Such immunometabolic remodeling is particularly relevant in the principal metabolic organs responsible for maintaining systemic glucose and lipid balance, ultimately leading to insulin resistance, β-cell dysfunction, and the onset of T2DM. The following section discusses how these dual functional modes of HDAC3 are translated into organ-specific immunometabolic abnormalities that drive the pathogenesis of T2DM.

## 4 HDAC3-driven immunometabolic dysregulation in diabetes

The core pathological features of T2DM include IR, chronic low-grade inflammation, and progressive β-cell failure. Adipose tissue, liver, skeletal muscle, and the intestine represent key metabolic organs that maintain glucose and lipid homeostasis, while immune cell infiltration and sustained release of inflammatory mediators act as major amplifiers of metabolic dysregulation (68). Among these metabolic organs, the intestine is uniquely positioned at the interface between host metabolism and the gut microbiota, integrating dietary nutrients, microbial metabolites, and epigenetic regulation to influence systemic metabolic homeostasis. This distinctive role also provides a conceptual framework for understanding how microbiota-derived metabolites modulate HDAC3 activity. Current evidence indicates that HDAC3 is hyperactivated in both metabolic and immune cells, positioning it as a central epigenetic hub that links inflammatory signaling to metabolic gene regulation, thereby driving systemic metabolic dysfunction (69, 70). As summarized in **Figure 5**, persistent HDAC3 activation drives a positive feedback loop in which inflammation promotes metabolic dysfunction. In addition, a previous study demonstrated that HDAC3 reprograms macrophage lipid metabolism, suppresses fatty acid oxidation, and promotes IL-1β-dependent inflammatory responses (71). Together, these findings support a self-reinforcing cycle whereby inflammation exacerbates metabolic dysfunction, while metabolic disturbances further amplify inflammatory signaling, ultimately contributing to the development and progression of multi-organ complications in T2DM.

**Figure 5:**
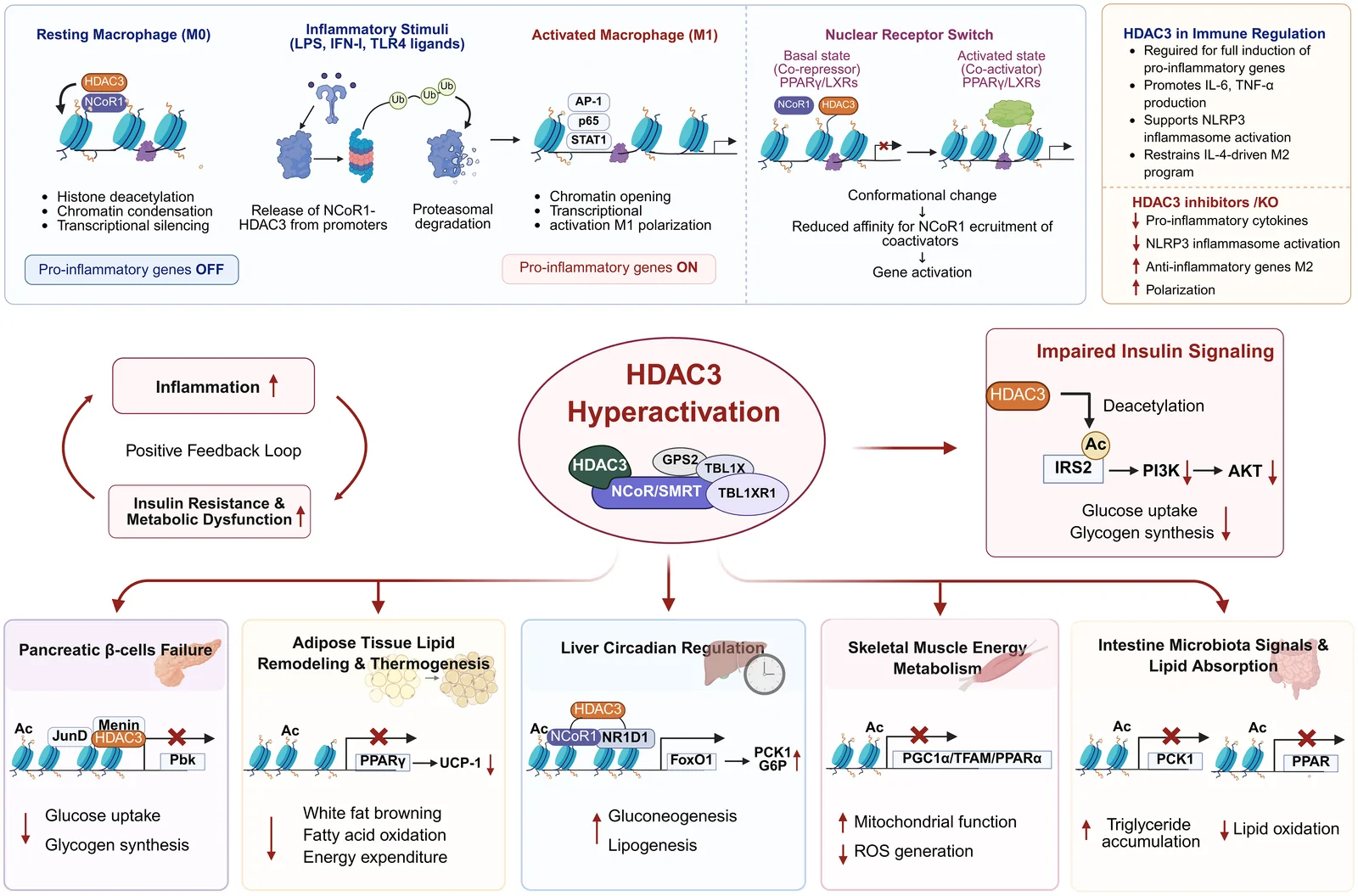
HDAC3-driven immunometabolic dysfunction in T2DM. The upper panel illustrates HDAC3-mediated macrophage polarization under inflammatory stimuli. In resting M0 macrophages, HDAC3-NCoR1 complex mediates histone deacetylation to silence pro-inflammatory genes. Stimulation triggers proteasomal degradation of HDAC3, chromatin relaxation and M1 macrophage activation, while HDAC3 also participates in nuclear receptor conformational switching to sustain inflammatory transcription. Excessive HDAC3 forms a positive feedback loop linking persistent inflammation and insulin resistance. Hyperactivated HDAC3 represses IRS2 via deacetylation to block downstream PI3K-Akt insulin signaling and reduce glucose utilization. In pancreatic β-cells, adipose tissue, liver, skeletal muscle and intestinal epithelium, HDAC3 epigenetically suppresses key metabolic transcription programs, disrupting insulin secretion, fat browning, hepatic glucose and lipid homeostasis, muscle mitochondrial function and intestinal lipid metabolism, collectively inducing systemic immunometabolic disorders of type 2 diabetes.

### 4.1 HDAC3-mediated β-cell failure

The inability of pancreatic β-cells to compensate for IR is a key determinant of T2DM progression. Inhibition of HDAC3 has been shown to markedly protect β-cells from both inflammatory and metabolic stress-induced damage (72). PDZ-binding kinase (PBK), a serine/threonine kinase that promotes β-cell proliferation, is transcriptionally repressed in high-fat diet (HFD)-induced T2DM models. Specifically, the transcription factor JunD recruits the menin-HDAC3 complex to the Pbk promoter, reducing histone H3 acetylation and suppressing Pbk expression, thereby blocking β-cell proliferation (73). Furthermore, proinflammatory cytokines can induce β-cell apoptosis, which is significantly mitigated by HDAC3-targeted inhibitors, although the precise molecular mechanisms underlying this protective effect remain to be fully elucidated (74, 75).

### 4.2 HDAC3-driven metabolic organ reprogramming and IR

#### 4.2.1 Adipose tissue: lipid remodeling and thermogenic control

Obesity is a major pathogenic driver of T2DM. HDAC3 expression in adipose tissue is strongly correlated with homeostasis model assessment of insulin resistance (HOMA-IR), circulating insulin levels, and high-sensitivity C-reactive protein (hs-CRP) (76).

Browning of white adipose tissue (WAT) enhances thermogenesis and fatty acid oxidation to support systemic glucose homeostasis (77). Adipose-specific loss or pharmacological inhibition of HDAC3 amplifies this browning program by increasing histone acetylation at the regulatory loci of Pparg, uncoupling protein 1 (Ucp1), and Ppara and by enhancing PPARγ transcriptional activity and stability, thereby promoting adipocyte differentiation and maturation, increasing whole-body energy expenditure, and ultimately improving metabolic homeostasis (78). Thus, under metabolic stress conditions, sustained HDAC3 activity may act as a barrier to adipose browning and metabolic improvement.

#### 4.2.2 Liver: circadian regulation of gluconeogenesis and lipid homeostasis

The liver is the principal source of fasting hyperglycemia. In hepatocytes, HDAC3 is rhythmically recruited to the hepatic genome in a circadian manner (79), where it cooperates with the NR1D1-NCoR1 complex to suppress lipogenesis and promote gluconeogenesis during the inactive phase, while exerting the opposite effects during the active phase (80).

Loss of HDAC3 leads to uncontrolled activation of lipogenic pathways and a re-routing of metabolic flux, shifting precursors away from gluconeogenesis toward lipid synthesis and sequestration. This metabolic rewiring ultimately reduces hepatic glucose output and improves systemic insulin sensitivity (81). During fasting, peroxisome proliferator-activated receptor γ coactivator 1 α (PGC1α) and forkhead box O1 (FoxO1) synergistically induce transcription of phosphoenolpyruvate carboxykinase 1 (PCK1) and glucose-6-phosphatase (G6Pase) to drive gluconeogenesis, and HDAC3 enhances this program by deacetylating FoxO1 (82). Pharmacological inhibition of HDAC3 diminishes FoxO1 activity and alleviates hyperglycemia in T2DM models (83).

Consistently, mice lacking NCoR1 are protected from diet-induced obesity and IR, further establishing the NCoR1-HDAC3 complex as a central regulatory node of hepatic metabolic homeostasis (80).

#### 4.2.3 Skeletal muscle: energy metabolism and inter-organ metabolic axis

Skeletal muscle is the primary tissue responsible for insulin-stimulated glucose uptake. Muscle-specific deletion of HDAC3 results in impaired glucose tolerance and IR (84), highlighting its essential role in maintaining muscle metabolic function. When HDAC3 enzymatic activity is abolished by mutations in the NCoR or SMRT corepressors, histone H3 becomes hyperacetylated at genes involved in branched-chain amino acid catabolism, including Bcat2, as well as at Ampd3, which encodes the rate-limiting enzyme of the purine nucleotide cycle. These animals display reduced force generation, enhanced fatigue resistance, increased fatty-acid oxidation, diminished glucose uptake during exercise, and upregulated expression of metabolic genes involved in branched-chain amino acid degradation (85). Pharmacological inhibition or knockdown of HDAC3 restores PPARα and fatty-acid oxidation gene expression, enhances mitochondrial oxidative phosphorylation, and reduces reactive oxygen species (ROS) production, thereby improving muscle energetic capacity (86).

#### 4.2.4 Intestinal epithelium: microbiota-derived metabolites and HDAC3-dependent metabolic regulation

The intestine represents a critical interface for nutrient absorption and for microbiota-host metabolic crosstalk. HDAC3 modulates histone acetylation in intestinal epithelial cells and integrates microbiota-derived signals to preserve intestinal metabolic homeostasis. Mice with an intestinal epithelial cell-specific deletion of HDAC3 exhibit increased energy expenditure, reduced adiposity, and improved glucose tolerance when challenged with HFD. Mechanistically, loss of HDAC3 releases the repression of PPAR-dependent transcriptional programs that drive lipid oxidation, leading to a broad remodeling of intestinal lipid metabolic profiles (87, 88). Collectively, these findings identify HDAC3 as a key epigenetic regulator of intestinal lipid oxidation and an important modulator of obesity susceptibility.

Emerging evidence further indicates that gut microbial dysbiosis reshapes intestinal HDAC3-dependent epigenetic regulation through alterations in microbiota-derived metabolites. Gut microbial dysbiosis reduces the production of short-chain fatty acids (SCFAs), thereby enhancing intestinal HDAC3 activity and decreasing H3K27 acetylation. Consequently, PPARγ-dependent transcription is suppressed, leading to upregulation of the lipid transporter CD36, increased intestinal lipid absorption, and ultimately systemic insulin resistance (89). These findings also provide a mechanistic basis for therapeutic strategies that enhance microbiota-derived SCFA production or directly target HDAC3. Dietary supplementation with the prebiotic inulin increases microbiota-derived SCFA production, thereby suppressing HDAC3 activity, enhancing histone H3K27 acetylation, and alleviating metabolic inflammation (90). Furthermore, exogenous supplementation of SCFAs, especially butyrate, improves metabolic disorders by modulating the GPR/HDAC3 signaling pathway, restoring intestinal barrier function, and attenuating inflammatory responses, further highlighting SCFAs as important endogenous regulators of HDAC3-mediated immunometabolic homeostasis (91). Taken together, these findings identify HDAC3 as a key molecular mediator linking gut microbiota -derived metabolites to intestinal metabolic homeostasis, providing a mechanistic foundation for microbiota-based and HDAC3-targeted therapeutic strategies discussed in Section 6.

### 4.3 HDAC3-mediated inflammatory amplification and disruption of insulin signaling

Accumulating clinical evidence supports the involvement of HDAC3 in the pathogenesis of T2DM. A community-based case-control study in China identified three single-nucleotide polymorphisms in HDAC3, namely rs11741808, rs2547547, and rs2530223, that are associated with an increased risk of T2DM (92). Consistent with this genetic association, both the expression and enzymatic activity of HDAC3 are markedly elevated in peripheral blood mononuclear cells from individuals with T2DM and correlate positively with inflammatory status, poor glycemic control, and impaired insulin sensitivity (9). Interestingly, adipose tissue from individuals with obesity exhibits reduced HDAC3 levels compared with that of normal-weight women, and HDAC3 mRNA expression and activity are negatively associated with inflammatory markers, obesity indices, and insulin levels, suggesting a complex and context-dependent role in metabolic dysregulation (76). Functionally, elevated HDAC3 not only impairs pancreatic β-cell function and promotes β-cell apoptosis (75), but also disrupts insulin signaling (72). Mechanistically, HDAC3 deacetylates insulin receptor substrate-2 (IRS2) and suppresses its transcription, thereby inhibiting the PI3K-AKT pathway and reinforcing a vicious cycle of inflammation and IR (93, 94).

Chronic low-grade inflammation is a major driver of IR. Macrophages play a pivotal role in the inflammatory mechanisms driving T2DM and its complications (95–97). In macrophages, HDAC3 exerts context-dependent regulatory effects on pro-inflammatory mediators across distinct macrophage activation states (22). In resting macrophages, the NCoR1-HDAC3 complex binds the promoters of pro-inflammatory genes and deacetylates local histones, thereby compacting chromatin and maintaining transcriptional silencing of inflammatory programs (98, 99).

Upon stimulation with LPS or type I interferons, the NCoR1-HDAC3 complex dissociates from promoters and undergoes ubiquitin-mediated degradation, allowing pro-inflammatory transcription factors such as STAT1, NF-κB, and AP-1 to access chromatin and initiate inflammatory gene transcription, thereby driving M1 polarization (98). Activation of Toll-like receptor 4 and related pathways can further induce conformational changes in nuclear receptors, including PPARγ and liver X receptors (LXRs), reducing their affinity for NCoR1 while enhancing interactions with co-activators. This shift promotes the displacement of the NCoR1-HDAC3 complex from pro-inflammatory promoters (100, 101).

HDAC3 plays a critical role in initiating inflammatory transcriptional programs while simultaneously constraining anti-inflammatory gene expression. RNA-seq analyses have confirmed that HDAC3 is required for the full induction of inflammatory genes (102). Inhibition of HDAC3 reduces the expression of cytokines such as interleukin-6 (IL-6) and TNF-α (103). Loss of HDAC3 also facilitates the IL-4-driven alternative activation program by modulating H3K9ac at genes such as Clec7a (104). Moreover, HDAC3 deficiency suppresses activation of the NOD-like receptor family pyrin domain containing 3 (NLRP3) inflammasome (105). Recent studies have further expanded the immunoregulatory functions of HDAC3 beyond inflammatory gene activation. In retinal ischemia-reperfusion models, myeloid-specific HDAC3 deletion enhanced macrophage-mediated efferocytosis and promoted tissue repair by activating the CD5L/CD36 pathway. These findings suggest that HDAC3 also regulates the resolution phase of inflammation and macrophage-mediated tissue remodeling (106, 107). In addition, selective HDAC3 inhibition has been shown to modulate macrophage polarization in neuropathy models by reducing pro-inflammatory macrophage responses and inflammatory cytokine production. Although these studies were not conducted in diabetic complications, they provide additional mechanistic evidence supporting the role of HDAC3 in macrophage-dependent inflammatory regulation (108). Taken together, these findings indicate that HDAC3 functions not only as a regulator of individual metabolic organs but also as a systemic coordinator of immunometabolic homeostasis. Persistent HDAC3 activation establishes a self-reinforcing cycle in which metabolic dysfunction, insulin resistance, and chronic inflammation perpetuate one another across multiple tissues. As this systemic immunometabolic imbalance persists, it creates a pathogenic milieu that increases the vulnerability of multiple organs to diabetic injury (109). Within this shared pathological context, HDAC3 subsequently drives tissue-specific molecular and cellular alterations through distinct downstream mechanisms, thereby contributing to the development of diabetic complications discussed in the following section.

## 5 HDAC3-driven shared mechanisms of diabetic complications

Building upon the systemic immunometabolic disturbances described above, sustained HDAC3 activation progressively drives tissue-specific pathological remodeling in multiple organs. By modulating transcriptional networks and chromatin accessibility, HDAC3 regulates inflammatory tone, mitochondrial function, and tissue remodeling, thereby driving the susceptibility of multiple organs to diabetic injury (**Figure 6**).

**Figure 6:**
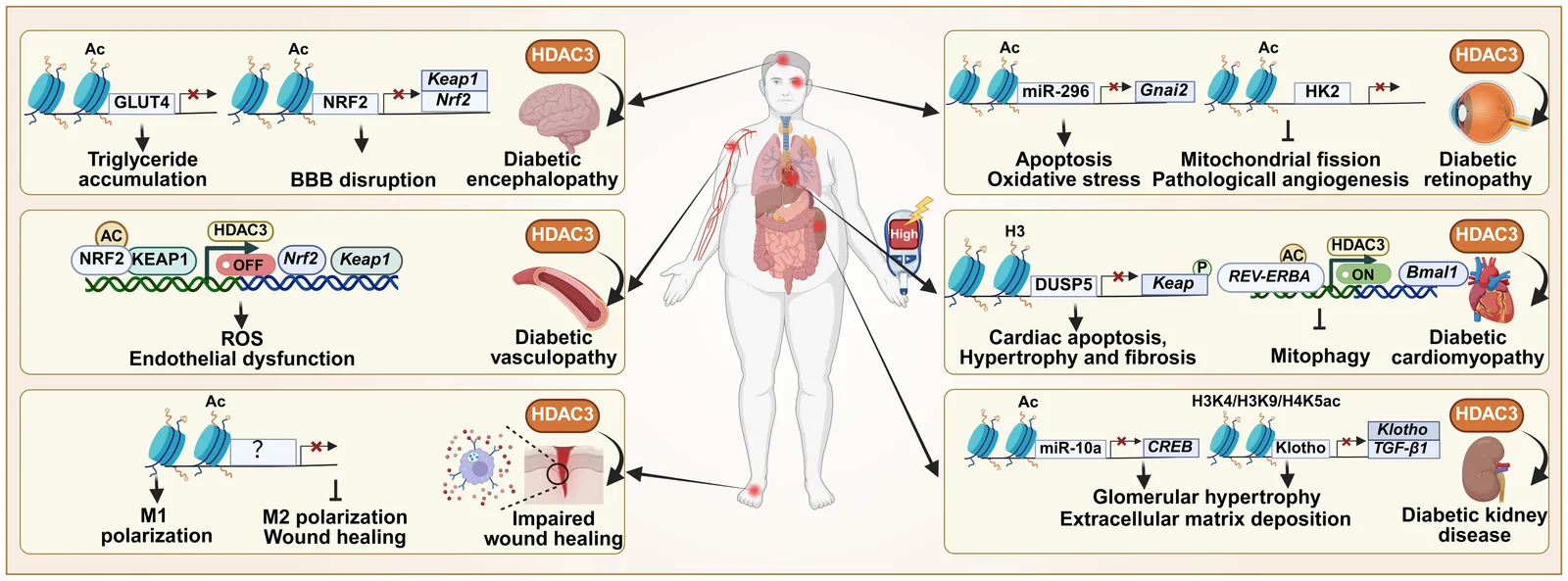
HDAC3-mediated pathogenic roles in diabetic complications. Hyperactivated HDAC3 under hyperglycemia mediates widespread epigenetic transcriptional suppression across diverse peripheral and central tissues. It broadly represses antioxidant, metabolic, anti-inflammatory and tissue-repair gene programs, disturbs macrophage polarization balance, and dysregulates glycolytic, circadian and fibrotic signaling cascades. These tissue-wide transcriptional disruptions collectively induce oxidative injury, inflammatory activation, vascular dysfunction and extracellular matrix accumulation, jointly facilitating the onset and progression of multiple systemic complications of T2DM.

### 5.1 Diabetic encephalopathy

Persistent hyperglycemia and systemic inflammation in T2DM progressively impair neuronal metabolic homeostasis, synaptic integrity, and cognitive function. A prominent early defect is reduced neuronal glucose uptake, characterized by diminished glucose transporter 4 (GLUT4) expression and heightened inflammatory signaling within the hippocampus (110). In human neurons, TNF-α-driven inflammatory activation suppresses solute carrier family 2 member 4 (SLC2A4, encoding GLUT4) and synaptic function-related genes (SYN1, TH, SYP), accompanied by decreased histone H3 or H4 acetylation, indicating a HDAC3-dependent transcriptionally repressive chromatin state. Pharmacological inhibition of HDAC3 effectively reverses these epigenetic and functional deficits (111).

Neural stem cells (NSCs) exhibit similar vulnerability: diabetes-induced HDAC3 upregulation restricts NSC differentiation, whereas HDAC3 knockdown restores neurogenic capacity (112). In parallel, HDAC3 disrupts blood-brain barrier (BBB) integrity by suppressing miR-200a expression, thereby enhancing Keap1-mediated inhibition of Nrf2 antioxidant signaling and downregulating tight-junction proteins, ultimately increasing barrier permeability (113). Collectively, these findings identify BBB disruption together with impaired neuronal metabolic homeostasis as the defining mechanisms through which HDAC3 promotes diabetic encephalopathy.

### 5.2 Diabetic cardiomyopathy

In the diabetic heart, HDAC3 is markedly activated and functions as a central epigenetic driver of myocardial dysfunction. HDAC3 regulates several pathogenic pathways that converge on oxidative stress, signaling imbalance, and impaired mitochondrial quality control. Consistent with the antioxidant regulatory mechanism described above, HDAC3 inhibition alleviates oxidative injury in diabetic hearts (114). Pharmacological inhibition of HDAC3 ameliorates diabetic cardiomyopathy, improving cardiac dysfunction, hypertrophy, and fibrosis while reducing oxidative stress, inflammation, and insulin resistance. Mechanistically, HDAC3-mediated histone H3 deacetylation suppresses dual specificity phosphatase 5 (DUSP5), thereby derepressing ERK1/2 signaling and promoting pathological cardiac remodeling. HDAC3 inhibition restores histone H3 acetylation at the DUSP5 promoter, suppressing ERK1/2 activation and providing sustained protection against diabetic cardiac injury (115).

Beyond this shared antioxidant mechanism, emerging evidence indicates that HDAC3 also regulates circadian rhythm and mitochondrial quality control, identifying circadian–mitochondrial coupling as a distinctive feature of diabetic cardiomyopathy. Mechanistically, diabetes-induced HDAC3 upregulation disrupts the oscillatory equilibrium between the circadian regulators nuclear receptor subfamily 1 group D member 1 (Rev-erbα) and brain and muscle ARNT-Like 1 (BMAL1), leading to defective mitophagy and heightened susceptibility to myocardial ischemia/reperfusion injury (MI/RI). Cardiac-specific HDAC3 knockdown restores Rev-erbα/BMAL1 rhythmicity, reactivates mitophagy, and confers substantial protection against diabetic MI/RI (116). Together, these findings identify circadian–mitochondrial coupling, rather than generalized oxidative stress, as the defining mechanism through which HDAC3 contributes to diabetic cardiomyopathy.

### 5.3 Diabetic vasculopathy

Diabetic vasculopathy (DV) is a chronic vascular complication of diabetes characterized by progressive endothelial dysfunction driven by oxidative stress and inflammation (117, 118). HDAC3 is highly activated in diabetes-associated endothelial injury and contributes to endothelial inflammation, ROS accumulation, and barrier disruption. Its upregulation in vascular endothelium and hippocampal/cortical tissues correlates with subclinical atherosclerosis (119). Consistent with its role in other diabetic tissues, HDAC3 impairs antioxidant defense and promotes oxidative stress in vascular endothelial cells (113, 120). Unlike other diabetic complications, the principal consequence of HDAC3 activation in the vasculature is persistent endothelial dysfunction, which promotes vascular remodeling and accelerates the progression of diabetic vascular disease. Collectively, persistent endothelial dysfunction and vascular remodeling represent the principal vascular-specific consequences of HDAC3 activation in diabetic vasculopathy.

### 5.4 Impaired diabetic wound healing

Delayed wound healing is a common complication of diabetes and arises from multiple pathological factors, among which chronic inflammation represents a major contributor. Accumulating evidence indicates that an imbalance in macrophage phenotypes plays a critical role in sustaining chronic inflammation in diabetic wounds (121). During normal wound healing, macrophages undergo a phenotypic transition from a pro-inflammatory (M1) state in the early phase to a pro-resolving, tissue-reparative (M2) state during later stages. However, this phenotypic transition is disrupted in diabetic wounds. Consequently, pro-inflammatory macrophages remain predominant, contributing to persistent inflammation and impaired tissue repair (122, 123). Epigenetic regulation, particularly histone modification, plays a pivotal role in controlling macrophage polarization. HDAC3 has been shown to sustain the M1 transcriptional program by modulating chromatin accessibility and nuclear receptor activity (65, 104). HDAC3 expression is markedly elevated in wounds of diabetic mice, accompanied by increased expression of M1-associated markers and reduced expression of genes characteristic of the M2 phenotype (124). Nevertheless, the key downstream targets through which HDAC3 mediates this regulatory process remain incompletely defined (125). Taken together, sustained M1 macrophage polarization and impaired inflammatory resolution represent the predominant mechanisms through which HDAC3 delays diabetic wound healing.

### 5.5 Diabetic kidney disease

HDAC3 is significantly upregulated in diabetic kidney disease and participates in fibrosis, inflammation, and loss of anti-aging signals (126). Deletion of HDAC3 reduces myofibroblast markers and markedly alleviates renal fibrosis. Mechanistically, HDAC3 recruits NCoR complex to the Klotho promoter, resulting in histone deacetylating and transcriptional repression of Klotho, while inflammatory signaling may further reinforce this process (127). The subsequent disruption of the Klotho-associated antifibrotic pathway and activation of profibrotic signaling represent a characteristic feature of HDAC3-mediated diabetic kidney injury. In parallel, HDAC3 suppresses miR-10a, thereby releasing cAMP response element-binding protein 1 (CREB1) and fibronectin expression and promoting extracellular matrix accumulation. HDAC3 overexpression is sufficient to induce diabetic-like renal alterations, whereas its knockdown restores miR-10a levels and mitigates these transcriptional and structural abnormalities (128). Collectively, these findings identify Klotho-associated profibrotic signaling, together with dysregulation of non-coding RNA networks, as the defining mechanisms through which HDAC3 promotes diabetic kidney disease.

### 5.6 Diabetic retinopathy

Hypoxia and oxidative stress are central drivers of diabetic retinopathy (DR), and HDAC3 is markedly upregulated in DR tissues. Pharmacological inhibition of HDAC3 attenuates retinal thinning and reduces the expression of vascular endothelial growth factor (VEGF) and glial fibrillary acidic protein (GFAP). It also enhances antioxidant capacity and protects retinal ganglion cells (RGCs) from apoptosis (129). HDAC3 also represses miR-296-5p, thereby weakening antioxidant defenses and aggravating vascular leakage (130).

Beyond its role in oxidative stress, emerging evidence indicates that HDAC3 is a critical regulator of endothelial metabolic reprogramming in DR. Endothelial metabolism is tightly linked to pathological neovascularization. Elevated glycolytic flux enhances aberrant angiogenesis (131). HDAC3 promotes mitochondrial fission and upregulates hexokinase 2 (HK2), thereby increasing glycolytic flux and driving pathological vessel growth, representing a key epigenetic node in endothelial metabolic reprogramming (132). Taken together, endothelial glycolytic reprogramming emerges as the defining mechanism that distinguishes HDAC3-mediated diabetic retinopathy from other diabetic complications.

### 5.7 Summary

Through coordinated regulation of transcriptional programs involved in metabolism and inflammation, HDAC3 promotes β-cell dysfunction, insulin resistance, metabolic organ remodeling, and ultimately the development of diabetic complications, thereby establishing a self-reinforcing immunometabolic network in T2DM.

Collectively, the current evidence indicates that HDAC3 contributes to the pathogenesis of T2DM and its complications through multiple molecular mechanisms. Notably, most studies have focused on the enzymatic deacetylase activity of HDAC3, including histone deacetylation-dependent transcriptional repression and the deacetylation of non-histone regulatory proteins. In contrast, direct evidence supporting non-enzymatic scaffolding functions of HDAC3 in diabetic models remains relatively limited and is currently restricted to a small number of pathways, particularly those involving co-repressor complexes and transcriptional regulatory assemblies. To better distinguish the current level of evidence supporting different functional modalities of HDAC3, we have summarized the representative mechanisms involved in T2DM and its complications and classified them according to whether they are supported by enzymatic or non-enzymatic mechanisms (**Table 1**).

**Table 1:** HDAC3 functions in T2DM and its complications: current evidence supporting enzymatic and non-enzymatic mechanisms.

Disease/Biological process	Representative mechanism	Functional mode
Macrophage inflammatory activation	NCoR1-HDAC3 dissociation, NF-κB/AP-1/STAT1 activation, M1 polarization	Enzymatic and non-enzymatic
IR	HDAC3-mediated deacetylation suppresses IRS2–PI3K–AKT signaling	Histone deacetylase enzymatic function
Pancreatic β-cell dysfunction	Menin-HDAC3 complex represses Pbk transcription and impairs β-cell function	Histone deacetylase enzymatic function
Adipose tissue remodeling and thermogenesis	HDAC3 represses PPARγ-UCP1 signaling and inhibits adipocyte browning	Histone deacetylase enzymatic function
Hepatic metabolic dysregulation	NCoR1-HDAC3-NR1D1 complex regulates FoxO1-mediated gluconeogenesis	Histone deacetylase enzymatic function
Skeletal muscle metabolic dysfunction	HDAC3 suppresses PGC1α/TFAM/PPARα signaling and mitochondrial metabolism	Histone deacetylase enzymatic function
Intestinal lipid absorption and microbiota signaling	HDAC3 represses PCK1 and PPAR-dependent metabolic programs	Histone deacetylase enzymatic function
Diabetic encephalopathy	HDAC3 suppresses GLUT4 and Nrf2 pathways, promoting BBB dysfunction and oxidative stress	Histone deacetylase enzymatic function
Diabetic vasculopathy	HDAC3 promotes Keap1-mediated repression of Nrf2 antioxidant signaling	Deacetylation of non-histone transcription factors
Diabetic wound healing	HDAC3 promotes M1 polarization and suppresses M2-mediated tissue repair	Histone deacetylase enzymatic function
Diabetic retinopathy	HDAC3-HK2 axis regulates glycolysis, mitochondrial fission and pathological angiogenesis	Histone deacetylase enzymatic function
Diabetic cardiomyopathy	DUSP5-ERK1/2 signaling axis	Histone deacetylase enzymatic function
	REV-ERBα-HDAC3-mediated circadian transcriptional regulation	Deacetylation of non-histone transcription factors
Diabetic kidney disease	HDAC3-mediated repression of Klotho and activation of TGF-β signaling	Histone deacetylase enzymatic function

## 6 Selective HDAC3 inhibitors

Over the past two decades, drug development targeting HDAC3 has advanced substantially, with multiple inhibitors being designed and validated across a variety of disease models. Several HDAC inhibitors have already been approved by the U.S. Food and Drug Administration (FDA) for clinical use, including belinostat, vorinostat, romidepsin, and panobinostat, and they are primarily used in oncology (133).

Given the diverse regulatory functions of HDAC3, therapeutic strategies targeting HDAC3 can be broadly categorized into three major approaches: synthetic small-molecule inhibitors that directly inhibit HDAC3 enzymatic activity, miRNA-based approaches that suppress HDAC3 expression at the post-transcriptional level, and naturally derived compounds that modulate HDAC3-associated signaling networks. These strategies differ substantially in their molecular targets, mechanisms of action, therapeutic potential, and translational challenges. Therefore, they are discussed separately below to provide a systematic comparison of current HDAC3-targeted interventions.

### 6.1 Synthetic small-molecule HDAC3 inhibitors

Most HDAC3 inhibitors are competitive inhibitors containing zinc-binding groups that chelate the catalytic zinc ion within the active site of HDAC3 (134). The most commonly employed zinc-binding groups are the ortho-aminoanilide and hydroxamic acid moieties.

Among these compounds, RGFP966 and BRD3308 are the most extensively characterized selective HDAC3 inhibitors and have demonstrated therapeutic potential in metabolic and inflammation-associated disorders. By inhibiting HDAC3-mediated deacetylation, these compounds regulate transcriptional programs involved in metabolism, inflammation, mitochondrial homeostasis, and tissue repair.

RGFP966 is one of the most extensively studied selective HDAC3 inhibitors in metabolic disease models. In insulin resistance models, RGFP966 restores insulin secretion-related gene expression and protects pancreatic β-cells from lipotoxic injury, improving glucose homeostasis (135). In diabetic retinopathy, RGFP966 suppresses HDAC3-dependent glycolytic reprogramming and mitochondrial dysfunction, thereby reducing retinal endothelial cell migration and pathological angiogenesis (132). Beyond metabolic disorders, RGFP966 has also shown protective effects in non-diabetic diseases, mainly through modulation of inflammatory responses, oxidative stress, and mitochondrial homeostasis (105, 136).

BRD3308 is another selective HDAC3 inhibitor with therapeutic potential in metabolic and inflammatory disorders. In diabetes models, BRD3308 suppresses HDAC3 activity and pancreatic inflammatory infiltration, thereby preserving β-cell survival and improving islet function (74). Additional studies in inflammatory injury models suggest that BRD3308 may exert broader protective effects through regulation of PPARγ signaling, inflammasome activation, and autophagy pathways (137, 138).

Other synthetic compounds have also been explored for HDAC3 modulation. Entinostat (MS-275), an orally available class I HDAC inhibitor, exhibits anticancer activity but lacks HDAC3 specificity, making its effects difficult to attribute solely to HDAC3 inhibition (139). Comparably, novel o-aminobenzamide-based class I HDAC inhibitors also show poor HDAC3 selectivity and simultaneously inhibit HDAC1/2/3; in colorectal cancer cells, they disrupt the G2/M checkpoint to induce cell-cycle arrest and apoptosis, and yield stronger *in-vivo* antitumor activity than entinostat even in the presence of short-term *in-vitro* autophagic compensation (140).

Despite promising preclinical findings, selective HDAC3 inhibitors remain largely restricted to experimental studies, and no HDAC3-specific inhibitor has been approved for the clinical treatment of T2DM or its complications. Their clinical translation is currently limited by insufficient isoform selectivity, potential off-target effects, inadequate pharmacokinetic and long-term safety data, and the inability to selectively modulate the non-enzymatic scaffolding functions of HDAC3. Future development of HDAC3 inhibitors should therefore focus on improving isoform selectivity, tissue specificity, and functional selectivity.

### 6.2 miRNA-based approaches targeting HDAC3 expression

Unlike small-molecule inhibitors that primarily suppress HDAC3 catalytic activity, miRNA-based strategies regulate HDAC3 at the gene-expression level through post-transcriptional mechanisms. By directly binding to complementary sequences within HDAC3 mRNA, miRNAs can reduce HDAC3 protein expression and subsequently modulate HDAC3-dependent epigenetic and transcriptional programs. This approach provides an alternative means of regulating HDAC3 function without directly targeting the highly conserved catalytic domain.

Several miRNAs have been identified as endogenous regulators of HDAC3 expression and have demonstrated therapeutic potential in different disease models, including neurological injury, cardiovascular disorders, and cancer. For example, miR-494 suppresses HDAC3 expression following acute ischemic stroke, resulting in increased H3K9 acetylation, reduced neuronal apoptosis, and improved neurological outcomes (141). Similarly, miR-193b-3p regulates HDAC3 expression in subarachnoid hemorrhage models, leading to enhanced acetylation of NF-κB p65 and attenuation of neuroinflammatory responses (142). In atherosclerosis models, miR-328-5p-mediated inhibition of HDAC3 increases ATP-binding cassette transporter A1 expression in macrophage-derived foam cells, thereby reducing lipid accumulation (143). In triple-negative breast cancer, miR-589-5p targeting of HDAC3 suppresses cancer cell proliferation and colony formation capacity (144).

Despite these promising findings, HDAC3-regulating miRNA strategies remain largely restricted to preclinical studies outside the metabolic disease field. To date, no HDAC3-targeting miRNA-based therapeutic approach has been developed or validated for type 2 diabetes mellitus (T2DM) or its complications, highlighting an important gap in the translational application of this strategy.

miRNA-based approaches offer sequence-specific regulation of HDAC3 expression without directly targeting its conserved catalytic domain. However, their clinical application is limited by challenges including inefficient tissue delivery, instability, immune activation, and off-target effects. Future advances in delivery systems and disease-specific miRNA networks will be critical for therapeutic translation.

### 6.3 Natural compounds regulating HDAC3-associated pathways

In addition to synthetic inhibitors and miRNA-mediated approaches, increasing evidence suggests that natural compounds modulate HDAC3-associated signaling networks and improve metabolic disorders. Unlike selective HDAC3 inhibitors, they generally regulate HDAC3 indirectly through multiple molecular pathways rather than directly targeting its catalytic domain. Jatrorrhizine is a bioactive constituent of Coptis chinensis and has shown therapeutic potential in diabetic peripheral neuropathy. It suppresses HDAC3-mediated recruitment of Atxn2l and restores NRG1-ErbB2-PI3K-AKT signaling. As a result, Schwann-cell myelination is promoted, leading to improved neural function in diabetic peripheral neuropathy mice (145). Other natural compounds have also demonstrated HDAC3-related protective effects in metabolic disease models. Puerarin alleviates diabetic osteoporosis by suppressing HDAC3-associated inflammatory and apoptotic signaling, thereby restoring the balance between osteogenesis and osteoclast activity (146). Astragaloside I protects against diabetic kidney disease through activation of the Klotho/TGF-β1/Smad signaling pathway (127), whereas arctigenin improves obesity-associated metabolic disorders by regulating the SCFA-GPR41/43-HDAC3-FOXP3 signaling axis (147). Collectively, these studies indicate that natural compounds exert protective effects through modulation of HDAC3-associated transcriptional and signaling networks rather than selective inhibition of HDAC3 enzymatic activity.

Natural compounds represent a valuable source of HDAC3 modulators, particularly for chronic metabolic diseases because of their multi-target regulatory effects. However, their clinical translation is limited by poor bioavailability, insufficient molecular specificity, and inadequate pharmacokinetic and safety evaluation. Many HDAC3-modulating compounds are derived from traditional Chinese medicine, providing a rationale for exploring combination strategies with selective HDAC3 inhibitors. In addition to plant-derived natural compounds, microbiota-derived metabolites have recently emerged as another important class of endogenous regulators of HDAC3, providing a mechanistic link between gut microbial homeostasis and host metabolism.

### 6.4 Microbiota-derived metabolites targeting HDAC3

Gut microbiota-derived metabolites have recently emerged as a distinct class of endogenous HDAC3 modulators, providing an alternative strategy for regulating HDAC3-dependent metabolic homeostasis. Among these metabolites, short-chain fatty acids (SCFAs), particularly butyrate, are the best-characterized regulators because they function as broad-spectrum HDAC inhibitors while maintaining intestinal immunometabolic homeostasis.

Consistent with the mechanisms discussed in Section 4.2.4, butyrate suppresses HDAC3 activity in intestinal epithelial cells and enhances histone acetylation, thereby improving β-cell function, glucose metabolism, intestinal barrier integrity, and metabolic inflammation (148). In addition to exogenous butyrate supplementation, increasing endogenous SCFA production through microbiota-targeted interventions, such as prebiotic supplementation or high-cellulose diet (90, 149), has also been shown to suppress HDAC3 activity and improve systemic metabolic homeostasis, supporting the therapeutic potential of microbiota-based strategies.

Unlike selective HDAC3 inhibitors, microbiota-derived metabolites regulate HDAC3 indirectly and exert broad biological effects that extend beyond epigenetic modulation. Their clinical application is further limited by inter-individual variability in gut microbiota composition, limited tissue specificity, rapid metabolism, and uncertain dose-response relationships. Therefore, combining microbiota-targeted interventions with selective HDAC3 inhibitors may represent a complementary strategy to simultaneously restore metabolic homeostasis and achieve precise epigenetic regulation in diabetic complications.

A summary of HDAC3-targeted interventions in T2DM and its complications is provided in **Table 2**, whereas HDAC3-targeted strategies investigated in non-diabetic disease models are summarized in **Supplementary Table 1** (56, 100, 126–129, 133-141, 162–167). Current HDAC3-targeted interventions include selective small-molecule inhibitors, miRNA-mediated regulation, natural compounds, and microbiota-derived metabolites. Although these strategies act through different mechanisms, most primarily regulate HDAC3 expression or deacetylase activity. Approaches targeting the non-enzymatic scaffolding functions of HDAC3 are still lacking. Limited tissue specificity, insufficient clinical validation, and potential off-target effects remain major barriers to clinical translation. Future studies should develop functionally selective HDAC3 modulators and evaluate combination strategies with traditional Chinese medicine-derived natural compounds or microbiota-targeted interventions for T2DM and its complications.

**Table 2:** HDAC3-targeted interventions in T2DM and its complications: molecular mechanisms, therapeutic effects, and current limitations.

Inhibitor	Models	Key molecular signaling pathways	Results	Experimental evidence	HDAC isoform selectivity	Defects
RGFP966	Insulin resistance	Relieves HDAC3-mediated epigenetic repression of the insulin secretion program and protects β-cells from lipotoxic damage (135)	Lowers blood glucose and promotes insulin secretion	NIT-1 β-cells + PA-induced apoptosis, low-dose STZ-induced prediabetic ICR mice	Highly selective HDAC3 inhibitor	Lack of clinical data, Lack of pharmacokinetic data, Insufficient selectivity (58, 105, 107, 132, 135, 136, 150–154)
	Diabetic retinopathy	Blocks HK2 upregulation, inhibits glycolysis, reduces mitochondrial fission, and reshapes the metabolic phenotype (132)	Attenuates retinal endothelial cell migration and pathological angiogenesis, thereby alleviating hypoxia/ischemia-induced pathological neovascularization	Bovine retinal endothelial cells + OGD/R, C57BL/6J OIR mouse pups		
BRD3308	Diabetes mellitus	Inhibits HDAC3 activity and suppresses pancreatic islet monocyte infiltration, thereby promoting β-cell survival and regeneration (74)	Improves β-cell morphology and quantity, and enhances β-cell function	INS-1E β-cells + PA/glucose, Female NOD/ShiLTJ mice	Preferential HDAC3 inhibitor	Lack of clinical data, Cross-inhibition of HDAC1/2 still exists, Weight gain (74, 137, 138)
Jatrorrhizine	Diabetic peripheral neuropathy	Promotes activation of Ataxin 2 like (Atxn2l) and the neuregulin 1 (NRG1)- erb-b2 receptor tyrosine kinase 2 (ErbB2)-PI3K-AKT signaling pathway, thereby enhancing Schwann cell myelination (145)	Improves neural structure and function of mice with diabetic peripheral neuropathy	Primary rat/mouse Schwann cells, db/db mice (DPN model)	Indirect HDAC3 modulator; isoform selectivity not established	Poor/low oral bioavailability, High-dose gastrointestinal toxicity risk (155, 156)
Puerarin	Diabetic osteoporosis	Blocks HDAC3-induced inflammatory and apoptotic signaling, improving the cellular oxidative-inflammatory microenvironment and restoring osteogenic/osteoclastic balance (146)	Prevents the progression of diabetic osteoporosis	Fructose-induced cell model; STZ-induced diabetic osteoporotic rats	Indirect HDAC3 modulator; isoform selectivity not established	Low oral bioavailability, Non-selective HDAC inhibitor (affects both HDAC1 and HDAC3), Multi-target phytoestrogen with incompletely elucidated mechanisms (146, 157)
Astragaloside I	Diabetic kidney disease	Upregulates Klotho, thereby inhibiting TGF-β1/Smad2/3 signaling and suppressing renal tubular and interstitial fibrosis (127)	Alleviates renal injury	SV40-MES-13 mesangial cells + high glucose; db/db mice	Indirect HDAC3 modulator; isoform selectivity not established	Its pharmacokinetic profile, isoform selectivity, and long-term safety as an HDAC3 inhibitor have not been established
Butyrate	Diabetes mellitus	Elevates H3/H4 and H3K27ac levels, reshaping the epigenetic status of genes related to insulin secretion and inflammatory responses (158)	Effectively reverses the dysfunction of pancreatic islet β-cells induced by IL-1β	INS-1/INS-1 832/13 cells + IL-1β	Pan-HDAC inhibitor	Pan-HDAC inhibitor, Short half-life requires high-dose or continuous administration, High-dose gastrointestinal side effects (159–161)

## 7 Discussion and perspective

HDAC3 plays critical roles in normal physiological processes, including the regulation of mammalian circadian rhythms and developmental programs (12), and also participates in metabolic adaptation by modulating the browning of WAT and regulating systemic energy homeostasis (168). In recent years, accumulating studies have linked dysregulated HDAC3 activity to a broad range of diseases, including neurodegenerative diseases (169), cancer (170), ischemic injury (107), fibrosis (46), and infection (171). Emerging clinical evidence further supports the relevance of HDAC3 to metabolic disorders. For example, markedly elevated HDAC3 enzymatic activity and protein expression have been observed in peripheral blood mononuclear cells from individuals with T2DM (9). Additionally, in adipose tissue from individuals with obesity, HDAC3 mRNA expression and enzymatic activity are negatively correlated with circulating insulin levels (76). This review systematically summarizes the structural characteristics of HDAC3 and its dual functional modes, encompassing both enzymatic deacetylation and non-enzymatic scaffold activities. We further discuss the mechanistic roles of HDAC3 in IR and the development of multiple diabetic complications, highlighting its function as a central epigenetic regulator linking metabolic stress with inflammatory signaling. In addition, we summarize the current therapeutic landscape of HDAC3-targeted inhibitors and discuss their potential translational value in metabolic disease intervention.

Despite the significant progress achieved in recent years, several key issues regarding HDAC3 research remain to be addressed. To provide a concise overview of unresolved questions and future research priorities, the major knowledge gaps in HDAC3-related diabetes research are summarized in **Table 3**. First, the sample sizes of existing clinical studies remain relatively small, and investigations of diabetic complications still rely predominantly on animal models. Therefore, future studies should include large-scale, multicenter prospective cohorts with longitudinal follow-up. Peripheral blood mononuclear cells, plasma, and, where available, disease-relevant tissue samples should be collected to quantify HDAC3 expression and activity together with metabolic and inflammatory biomarkers. Longitudinal analyses should determine whether these markers predict glycemic progression, diabetic complications, and therapeutic responses. Second, although numerous studies have demonstrated that HDAC3 participates in the regulation of gene expression, its precise regulatory loci and interaction networks remain incompletely defined in many physiological and pathological contexts. Moreover, current mechanistic studies investigating the immunometabolic roles of HDAC3 in T2DM and its complications have focused predominantly on macrophages, whereas its functions in other immune cell populations remain poorly understood. Future studies should integrate ChIP-seq or CUT&Tag with single-cell transcriptomic and chromatin accessibility analyses in disease-relevant tissues to identify cell type-specific HDAC3 binding sites and downstream target genes. Candidate targets should then be validated by genetic perturbation and functional assays. Third, although increasing evidence supports the non-enzymatic scaffolding functions of HDAC3, most mechanistic insights have been derived from cellular and experimental models. Direct evidence demonstrating the relevance of these mechanisms in human diabetic tissues remains limited. Future studies should integrate patient-derived samples with spatial omics and quantitative protein-interaction analyses to identify disease-relevant HDAC3 complexes. Key interactions should be validated by co-immunoprecipitation and proximity-labeling approaches, followed by structural characterization where feasible. Finally, current studies on HDAC3 inhibitors primarily focus on suppressing its enzymatic activity and are often developed for multiple diseases. Pharmacological strategies targeting the non-enzymatic functions of HDAC3 remain relatively limited. Future drug development should further improve HDAC3 isoform selectivity and explore compounds that disrupt disease-relevant protein–protein interactions or scaffolding functions while preserving physiological HDAC3 activity. Another promising direction is the development of combination therapies targeting HDAC3. Selective HDAC3 inhibitors provide precise epigenetic regulation, whereas natural products derived from traditional Chinese medicine exert complementary effects on inflammation, fibrosis, and metabolic dysfunction. Their combination may enhance therapeutic efficacy in diabetic complications. Microbiota-targeted interventions that increase endogenous SCFA production may further complement HDAC3-targeted therapies. Future studies should validate these strategies in preclinical and clinical settings. Overall, with the rapid development of multi-omics technologies and advances in precision drug design, a systematic elucidation of the tissue-specific regulatory mechanisms of HDAC3, together with the development of more selective targeted interventions, may provide new theoretical insights and therapeutic avenues for the prevention and treatment of diabetes and its multi-organ complications.

**Table 3:** Current knowledge gaps and future research priorities in HDAC3-related diabetes research.

Knowledge gap	Current evidence	Future research direction
Limited clinical validation of HDAC3-associated biomarkers	Clinical studies mainly report associations between HDAC3 levels/activity and T2DM-related metabolic features, large-scale longitudinal cohorts are lacking.	Establish multicenter longitudinal cohorts using blood and tissue samples
Limited understanding of HDAC3 regulation in immune cell subsets beyond macrophages	Current studies mainly focus on macrophages	Apply single-cell multi-omics to identify HDAC3 targets in different cell populations
Limited human tissue evidence for HDAC3-mediated molecular mechanisms	Mechanistic evidence for HDAC3 functions is mainly derived from cellular and animal models; human tissue validation remains limited.	Integrate patient-derived tissues with multi-omics and protein-interaction analyses to define disease-relevant HDAC3 regulatory mechanisms, including non-enzymatic scaffolding functions.
Lack of clinical-stage HDAC3-specific inhibitors	HDAC3-targeting compounds remain largely at the preclinical stage.	Improve HDAC3 isoform selectivity, pharmacokinetic properties, safety profiles, and tissue targeting to facilitate clinical translation.

## Glossary

ACE2: Angiotensin-Converting Enzyme 2
AIM2: Absent In Melanoma 2
ATXN2l: Ataxin 2 like
AT2: Alveolar Type 2
BBB: Blood-Brain Barrier
BMAL1: Brain And Muscle ARNT-Like 1
CAF: Cancer Associated Fibroblasts
CAK: CDK Activating Kinase
CC: Coiled-Coil
CDK: Cyclin Dependent Kinase
CHD: Calponin Homology Domain
ChIP-seq: Chromatin Immunoprecipitation Sequencing
CPT1A: Carnitine Palmitoyltransferase 1A
CRM1: Chromosomal Region Maintenance 1
CREB1: cAMP Response Element-Binding Protein 1
CTD: C-Terminal Domain
CUT&Tag: Cleavage Under Targets And Tagmentation
DA: Deacetylase Activity
DADs: Deacetylase-Activation Domains
DBD: DNA-Binding Domain
DSS: Dextran Sulfate Sodium
DUSP5: Dual Specificity Phosphatase 5
E-Cadherin: Epithelial Cadherin
EMT: Epithelial to Mesenchymal transition
ERBB2: ERB-Bb2 Receptor Tyrosine Kinase 2
eRNA: Enhancer RNA
EZH2: Enhancer Of Zeste Homolog 2
5-FU: 5-Fluorouracil
FOXA2: Forkhead Box A2
FOXO1: Forkhead Box O1
FOXP3: Forkhead Box P3
GABP: GA-Binding Protein
GBP5: Guanylate-Binding Protein 5
GFAP: Glial Fibrillary Acidic Protein
GLUT4: Glucose Transporter 4
GPX4: Glutathione Peroxidase 4
GSDMD: Gasdermin D
G6Pase: Glucose-6-Phosphatase
HATs: Histone Acetyltransferases
HDAC3: Histone Deacetylase 3
HFD: High-Fat Diet
HK2: Hexokinase 2
HMGB1: High Mobility Group Box 1
HOMA-IR: Homeostasis Model Assessment Of Insulin Resistance
hs-CRP: High-Sensitivity C-Reactive Protein
IL-1β: Interleukin-1 Beta
IL-6: Interleukin-6
IR: Insulin Resistance
IRS2: Insulin Receptor Substrate-2
KLF5: Kruppel-Like Factor 5
LPS: Lipopolysaccharide
LXRs: Liver X Receptors
MI/RI: Myocardial Ischemia/Reperfusion Injury
NF-κB: Nuclear Factor Kappa B
NCoR: Nuclear Receptor Corepressor
NR: Nuclear Receptor
Nrf1: Nuclear Factor Erythroid 2-Like 1
Nrf2: Nuclear Factor Erythroid 2-Related Factor 2
NRG1: Neuregulin 1
NSCs: Neural Stem Cells
OPG: Osteoprotegerin
PBK: PDZ-Binding Kinase
PCK1: Phosphoenolpyruvate Carboxykinase 1
PGC1α: Peroxisome proliferator-activated receptor γ coactivator 1-alpha
PPARγ: Peroxisome Proliferator-Activated Receptor Gamma
RANKL: Receptor Activator Of Nuclear Factor κB Ligand
Rev-erbα: Regulators Nuclear Receptor Subfamily 1 Group D Member 1
RGC: Retinal Ganglion Cell
ROBO4: Roundabout Guidance Receptor 4
ROS: Reactive Oxygen Species
SMRT: Silencing Mediator Of Retinoid And Thyroid Hormone Receptors
SLC2A4: Solute Carrier Family 2 Member 4
STAT: Signal Transducer and Activator of Transcription Proteins
T1DM: Type 1 Diabetes Mellitus
T2DM: Type 2 Diabetes Mellitus
TGF-β1: Transforming Growth Factor-β1
TLR4: Toll-Like Receptor 4
TNF-α: Tumor Necrosis Factor Alpha
Ucp1: Uncoupling Protein 1
VEGF: Vascular Endothelial Growth Factor
WAT: White Adipose Tissue
ZEB1: Zinc finger E-box binding homeobox 1.
