# Supplementary material for: HDAC3-driven immunometabolic reprogramming in type 2 diabetes and its complications: from enzymatic deacetylation to non-enzymatic scaffolding
Source: Front Immunol. 2026 Sep 23;17:1890366. doi: 10.3389/fimmu.2026.1890366 (PMC13643520; doi:10.3389/fimmu.2026.1890366)
Supplement: Supplementary file 1 [file Table1.docx]

**Table S1** HDAC3-targeted interventions in non-diabetic disease models and their molecular mechanisms and therapeutic effects

| **Inhibitor** | **Models** | **Key Molecular Signaling Pathways** | **Results** |
| --- | --- | --- | --- |
| RGFP966 | Ischemic stroke | Enhances STAT1 acetylation and subsequently attenuates STAT1 phosphorylation, thereby inhibiting the absent in melanoma 2 (AIM2) inflammasome (56) | Suppresses inflammation to alleviate ischemic stroke |
|  | Acute kidney injury progressing to chronic kidney disease | Relieves HDAC3-mediated inhibition of the Kruppel-like factor 5 (KLF5)-glutathione peroxidase 4 (Gpx4) axis, thereby restoring GPX4 expression and reducing ferroptosis (133) | Improves renal fibrosis and associated renal function decline |
|  | Renal fibrosis | Inhibits the binding of NCoR-NF-κB complexes to the Klotho promoter, thereby promoting Klotho transcription (134) | Alleviates renal fibrotic injury |
|  | Ischemic retinopathy | Relieves HDAC3-mediated inhibition of reparative phagocytosis, thereby enhancing macrophage-mediated clearance of apoptotic cells (135) | Reduces inflammation and protects retinal neurons and vascular integrity |
|  | Acute lung injury | Blocks HDAC3-mediated deacetylation of FOXO1, inhibits aberrant activation of the FOXO1-ROCK1 axis, and restores mitochondrial quality control in alveolar type 2 (AT2) cells (136) | Maintains epithelial barrier structure and function, and ameliorates sepsis-induced lung injury |
|  | Traumatic brain injury | Activates the Nrf2 antioxidant pathway and inhibits the high mobility group box 1 (HMGB1)/toll-like receptor 4 (TLR4)-mediated NLRP3 inflammasome pathway (137) | Reduces neuronal damage, apoptosis, and oxidative-stress-induced neuroinflammation |
|  | Allergic rhinitis | Blocks HDAC3-mediated inflammatory amplification and reduces goblet cell hyperplasia (138) | Alleviates allergic symptoms and suppresses goblet cell hyperplasia |
|  | Inflammatory bowel disease | Reduces guanylate-binding protein 5 (GBP5) expression, inhibits the NLRP3 inflammasome, and alleviates inflammatory responses (100) | Reduces the disease severity and tissue damage in Dextran Sulfate Sodium (DSS)-induced colitis |
|  | Neuropathic pain | Enhances the transcriptional activity of forkhead box A2 (FOXA2) and upregulates carnitine palmitoyltransferase 1A (CPT1A), improving lipid metabolism and mitochondrial homeostasis (139) | Restores microglial phagocytic function, alleviates aberrant synaptic pruning and neuroinflammatory responses, and ultimately reduces neuropathic pain |
| BRD3308 | Intraventricular hemorrhage | Activates the PPARγ signaling pathway, inhibits NLRP3 inflammasome activation, and downregulates caspase-1-mediated cleavage of gasdermin D (GSDMD) pyroptosis (140) | Reduces neuroinflammation, neuronal loss, and microglial activation, ultimately improving neurobehavioral function |
|  | Sepsis-induced acute lung injury | Elevates H3K27 acetylation and upregulates ATG5-mediated autophagy, reducing ROS accumulation and inhibiting NLRP3-driven pyroptosis (141) | Alleviates inflammation and tissue damage in sepsis-induced acute lung injury |
| Arctigenin | Obesity | Inhibits HDAC3 activity, and repairs the intestinal barrier function via the SCFA-GPR41/43-HDAC3- forkhead box P3 (FOXP3) axis, activating the hepatic GPR-AMPK pathway (151) | Alleviates inflammation and improves glycolipid metabolism, thereby counteracting obesity and metabolic disorders |
| Entinostat | Bladder cancer | Promotes H3K18la-mediated DHRS2 expression and  nuclear translocation to suppress the AKR1C3-androgen axis (166) | Overcomes cisplatin resistance in bladder cancer |
|  | Hepatocellular carcinoma | Increases NTF3 expression and activates the p75NTR-JNK/p38 signaling pathway (167) | Promotes apoptosis of hepatocellular carcinoma cells and inhibits their proliferation |
| miR-494 | Acute ischemic stroke | Suppresses HDAC3 expression through post-transcriptional regulation, increases acetylation of H3K9, and reduces neuronal apoptosis (126) | Mitigates acute ischemic brain injury and attenuates cerebral atrophy |
| miR-193b-3p | Subarachnoid hemorrhage | Increases the acetylation level of NF-κB p65 and attenuates neuroinflammatory signaling (127) | Mitigates neurobehavioral deficits and neuroinflammatory responses following subarachnoid hemorrhage |
| miR-328-5p | Atherosclerosis | Increases the level of ATP-binding cassette transporter A1 in foam cells derived from THP-1 macrophages (128) | Attenuates lipid accumulation in foam cells |
| miR-589-5p | Triple-negative breast cancer | Reduces the viability, proliferative capacity, and colony-forming ability of breast cancer cells by targeting HDAC3 (129) | Inhibits the proliferative activity of breast cancer cells |
| Tanshinone IIA | Skin cancer | Enhances transcriptional activation of Nrf2 and stimulates the Nrf2-dependent antioxidant pathway (168) | Suppresses the malignant transformation of JB6 cells |
| Kaempferol | Myocardial ischemic injury | Relieves HDAC3-mediated inhibition of Nrf2 and enhances Nrf2 acetylation, thereby initiating the antioxidant transcriptional program (169) | Effectively alleviates oxidative stress and myocardial injury |
| Pterostilbene | Ischemic stroke | Promotes acetylation of Nrf1 at K105/K139, inhibits the interaction between Nrf1 and p65, and reduces inflammation (47) | Suppresses the inflammatory activation of microglia |
| Juglanin | Chronic kidney disease | Blocks the nuclear translocation of NF-κB/HDAC3, inhibiting inflammation and lipid deposition (170) | Improves metabolic disorders, renal injury, and fibrosis |
| Silymarin | Lung cancer | Reduces ZEB1 expression, inhibits E-cadherin, and restores miR-203 levels (171) | Inhibits the metastatic potential of lung cancer |
